# Neurokinin-1 Receptor Signalling Impacts Bone Marrow Repopulation Efficiency

**DOI:** 10.1371/journal.pone.0058787

**Published:** 2013-03-14

**Authors:** Alexandra Berger, Catherine Frelin, Divya K. Shah, Patricia Benveniste, Robert Herrington, Norma P. Gerard, Juan-Carlos Zúñiga-Pflücker, Norman N. Iscove, Christopher J. Paige

**Affiliations:** 1 Ontario Cancer Institute, University Health Network, Toronto, Ontario, Canada; 2 Department of Medical Biophysics, University of Toronto, Toronto, Ontario, Canada; 3 Department of Immunology, University of Toronto, Toronto, Ontario, Canada; 4 Sunnybrook Research Institute, Toronto, Ontario, Canada; 5 Ina Sue Perlmutter Laboratory, Children's Hospital, Harvard Medical School, Boston, Massachusetts, United States of America; Emory University, United States of America

## Abstract

Tachykinins are a large group of neuropeptides with both central and peripheral activity. Despite the increasing number of studies reporting a growth supportive effect of tachykinin peptides in various *in vitro* stem cell systems, it remains unclear whether these findings are applicable *in vivo*. To determine how neurokinin-1 receptor (NK-1R) deficient hematopoietic stem cells would behave in a normal *in vivo* environment, we tested their reconstitution efficiency using competitive bone marrow repopulation assays. We show here that bone marrow taken from NK-1R deficient mice (*Tacr*1^−/−^) showed lineage specific B and T cell engraftment deficits compared to wild-type competitor bone marrow cells, providing evidence for an involvement of NK-1R signalling in adult hematopoiesis. Tachykinin knockout mice lacking the peptides SP and/or HK-1 (*Tac1* ^−/−^, *Tac4* ^−/−^ and *Tac1* ^−/−^/*Tac4* ^−/−^ mice) repopulated a lethally irradiated wild-type host with similar efficiency as competing wild-type bone marrow. The difference between peptide and receptor deficient mice indicates a paracrine and/or endocrine mechanism of action rather than autocrine signalling, as tachykinin peptides are supplied by the host environment.

## Introduction

Tachykinins are a group of small neuropeptides, which share the C-terminal motif FXGLM-NH_2_. In mouse, *Tac1* encodes substance P (SP) and neurokinin A (NKA) through alternative splicing, *Tac2* produces neurokinin B (NKB), and *Tac4* encodes hemokinin-1 (HK-1). Tachykinins mediate their actions through three G-protein coupled receptors, neurokinin-1 receptor (NK-1R, *Tacr1*), NK-2R (*Tacr2*) and NK-3R (*Tacr3*). While each ligand can interact with all receptors with varying affinity, SP and HK-1 are the preferred, endogenous ligands for NK-1R [1], [2], NKA preferentially binds to NK-2R, and NKB to NK-3R [3].

Stem cells are characterized by their ability to self-renew as well as to generate differentiated progeny [4], [5], and thus are regarded as a promising tool for regenerative and transplantation medicine. Embryonic stem cells are pluripotent and able to differentiate into almost all cell types, whereas adult stem cells such as neuronal stem cells or hematopoietic stem cells are more restricted in their potential and are specialized to differentiate into certain cell lineages. For example during adult hematopoiesis, hematopoietic stem cells produce lymphoid and myeloid cells to maintain a steady supply of immune cells.

Several studies have recently demonstrated expression and/or growth promoting functions for neuropeptides in various *in vitro* stem cell systems, including galanin [6], neuropeptide Y [7] and substance P [8], [9], [10], [11]. In human bone marrow, SP and NKA have been described to exert opposing effects on hematopoiesis *in vitro* via NK-1R and NK-2R [12], [13]. Shahrokhi et al. showed a significant improvement of expansion of human cord blood stem cells when grown in the presence of SP [11]. We observed increased cell proliferation rates of embryonic (E)14 stem cells when grown in the presence of SP and/or HK-1 (unpublished observation). Hong et al. reported that SP was capable of inducing HSC to migrate to the blood and engage in tissue repair *in vivo* [14]. Despite the mounting evidence for the involvement of tachykinins in stem cell biology, limited *in vivo* data are available.

We wanted to expand on our recent study where we have described the generation of the *Tac4* ^−/−^ mouse and reported a 2-fold increase in the pro B cell population [15]. Consistent with our *in vivo* data, *in vitro* cultures derived from *Tac4* ^−/−^ long-term reconstituting hematopoietic stem cells contained significantly higher absolute numbers of pro B cells compared to wild-type cultures. Addition of HK-1 to these cultures established from long-term reconstituting hematopoietic stem cells lead to a significant decrease of *de novo* generated pro B cells, suggesting an inhibitory role for HK-1 and its receptors in hematopoiesis [15].

In this study we sought to determine how neurokinin-1 receptor deficient hematopoietic stem cells would behave in a normal environment. We therefore examined whether total bone marrow isolated from neurokinin-1 receptor deficient mice (*Tacr1* ^−/−^) would repopulate a lethally irradiated host with similar efficiency as wild-type bone marrow. Tachykinin knockout mice lacking the peptides SP and/or HK-1 (*Tac1* ^−/−^, *Tac4* ^−/−^ and *Tac1* ^−/−^/*Tac4* ^−/−^ mice) were also included in this long-term bone marrow reconstitution study to determine whether a hematopoietic stem cell lacking tachykinin peptides would perform differently from a wild-type stem cell.

## Materials and Methods

### Animals

The following animals were used in this study: *Tac4* ^−/−^, N8, [15]; *Tac1* ^−/−^, N10 [16] (The Jackson Laboratory, Bar Harbor, ME, USA); *Tac1* ^−/−^/*Tac4* ^−/−^ mice, N9, [17] and *Tacr*1^−/−^ mice, N10 [18]. C57BL/6 breeding pairs were purchased from the Jackson Laboratory (Bar Harbor, ME, USA), bred in-house and used as wild-type controls. Mice were housed under specific pathogen-free conditions at a constant temperature (22°±2°) on a 12-hour light-dark cycle (light cycle: 6 am–6 pm). Food and water were available *ad libitum*. Animal experiments were approved by the University Health Network Animal Care Committee and performed in compliance with current institutional guidelines. All of the genetically altered mouse strains (*Tac4* ^–/–^, *Tac1* ^–/–^, *Tacr1* ^–/–^, *Tac1* ^–/–^/*Tac4* ^–/–^) are genotyped on a regular basis including time points before and after the experiments reported in this manuscript. The results of one such analysis, which was done shortly after the experiments reported here, are shown in Fig. S1.

### Bone marrow transplants and competitive *in vivo* repopulation assays

Bone marrow was harvested by flushing femurs and tibias with injection media (IBSS containing 0.5% FBS and 3% kit ligand conditioned media) [4], and the cell suspension was filtered and counted. For the competitive *in vivo* repopulation, bone marrow cells from a donor mouse (C57BL/6, *Tac1* ^–/–^, *Tac4* ^–/–^, *Tac1* ^–/–^/*Tac4* ^–/–^ or *Tacr1* ^–/–^; *Ly5.2-Gpi1^b/b^*) were mixed 1∶1 with bone marrow cells from a wild-type mouse of the same strain as the recipient mouse (C57BL/6, *Ly5.1-Gpi1^a/a^*). The mixture of cells was intravenously injected into lethally irradiated (9 Gy, Cs^137^) 8–12 week old *Ly5.1-Gpi1^a/a^* recipient mice, with each recipient receiving 1×10^6^ donor (*Ly5.2-Gpi1^b/b^*) and 1×10^6^ competitor (*Ly5.1-Gpi1^a/a^*) bone marrow cells. The contribution of donor to recipient blood T cells (CD3^+^), B cells (B220^+^), myeloid cells (Mac1/Gr1^+^) and red blood cells (Gpi1) was assessed 8, 16, 24 and 32 weeks post bone marrow transplantation by flow cytometry. To establish the proportion of *Ly5.2-Gpi1^b/b^* ^+^ red blood cells, erythrocyte Gpi1 isoforms were resolved on sepharose membranes and quantitated as described [5]. Four independent reconstitution experiments were performed. Up to five individual mice per strain were injected in each experiment. Each knockout strain was graphed versus the wild-type strain and differences were analyzed for each time point separately using Student's t-test (Prism Graph Pad).

### FACS analysis of bone marrow and thymus

Bone marrow was flushed with sterile PBS containing 3% fetal calf serum (FCS). Thymus was forced through a 40 µm nylon cell strainer (BD Biosciences, Mississauga, Canada) in sterile PBS containing 3% FCS. Cells were centrifuged for 5 min at 1250 rpm. After red blood cell lysis, cells were resuspended in PBS with 3% FCS. Cells were stained for 30 min at 4°C with a combination of antibodies commonly used for analysis of immune cells. Antibodies were directly conjugated to FITC, PE, biotin or APC and were purchased from BD Biosciences (Mississauga, Canada). PerCP Streptavidin (Streptavidin-Peridinin Chlorophyll-a Protein) was used to stain cells in combination with biotinylated primary antibodies. FACS analysis was performed on FACS Calibur (BD Biosciences, Mississauga, Canada) and data analysis was conducted using Cell Quest Pro. All experiments were performed using female mice, 8–12 weeks old. Every FACS experiment included age-matched sets of knockout and wild-type control mice.

### *In vitro* B cell experiments

For proliferation assays, bone marrow cells were seeded into B cell media (Opti-MEM with 10% non-heat inactivated FCS (Invitrogen, Carlsbad, CA, USA), 1×Penicillin/Streptomycin and 5.5×10^−5^ M 2-Mercaptoethanol) containing the appropriate growth factors (proliferation assay bone marrow: 5×10^4^ cells/96 well, growth factors/stimulants: no stimulation, 1 ng/ml IL-7, IL-3 (supernatant, 1∶100). Plates were pulsed on day four with ^3^H-thymidine (0.5 µCi/well), incubated at 37°C for 6 hours and harvested onto Uni-Filter-96, GF/C plates (Perkin Elmer, Shelton, CT, USA) using a plate harvester. The filter plates were dried, scintillation fluid was added and plates were measured using a Scintillation counter.

IL-7 frequency analysis was carried out by plating bone marrow cells in 200 µl B cell media containing 1 ng/ml IL-7 in 96 well plates (cell density: 100, 200, 400 and 800 cells/well). Wells were scored for the frequency of IL-7 responsive cells on day seven as previously described [19].

### Hematopoietic progenitor cell isolation, OP9 co-cultures and flow cytometry

Hematopoietic progenitor cells from LSK bone marrow cells were sorted by flow cytometry for CD117^+^ Sca1^+^ Lin^−^ (CD4^−^ CD8^−^ B220^−^ CD11b^−^ CD19^−^) cells, using either FACS-DIVA (BD-Biosciences) or FACS-Aria (BD-Biosciences) flow cytometers, and sorted cells were determined >99% pure by post-sort analysis. 1×10^4^ hematopoietic progenitor cells were seeded onto near confluent OP9 cells [20] in 6-well plates in co-culture with αMEM supplemented with 15% FBS (Gibco or Hyclone) and 1×Penicillin/Steptomycin (Invitrogen). Cytokines, human recombinant Flt3 ligand (1 ng/ml, R&D systems) and mouse IL-7 (5 ng/ml, Peprotech), were added to the co-cultures. Cells were harvested on day 7 for developmental progression and cellularity analysis or further passaged onto fresh plates of near confluent OP9 cells for analysis on days 10, 14 and 18.

Cells were stained using combinations of directly conjugated antibodies obtained from BD Pharmingen or eBiosciences. Flow cytometry analysis was performed on the FACS Calibur (Becton Dickinson Biosciences). Events were collected using CellQuest software and data analyzed using FlowJo software (Tree Star Inc.). Live cells were gated according to their Forward Scatter and Side Scatter profiles and gated negatively on propidium iodide (pI) staining. CD45 was used to gate lymphocyte populations and exclude OP9 cells. Data are representative of 2–3 experiments.

## Results and Discussion

An increasing number of *in vitro* studies has recently described positive effects such as enhanced proliferation of stem cells grown in the presence of tachykinins [8], [9], [10], [11], suggesting a role for tachykinins in stem cell biology. One of the goals of this study was to test whether a role for tachykinins in stem cell biology could be confirmed *in vivo*.

To examine how neurokinin-1 receptor deficient hematopoietic stem cells would behave in a normal environment, we tested the reconstitution efficiency of bone marrow taken from *Tacr1* ^-/-^ mice in a series of *in vivo* competitive repopulation assays into lethally irradiated recipient mice. Donor bone marrow cells (C57/BL/6; *Tacr1* ^—/—^; *Ly5.2-Gpi1^b/b^*) were mixed 1∶1 with bone marrow cells of a competing donor (C57BL/6, *Ly5.1-Gpi1^a/a^*) and transplanted into a lethally irradiated host (*Ly5.1-Gpi1^a/a^*). In order to examine long term reconstitution efficiency of the *Ly5.2-Gpi1^b/b^* stem cell population, blood was taken at 8, 16, 24 and 32 weeks after the initial transplant and the ratio of *Ly5.2-Gpi1^b/b^* to *Ly5.1-Gpi1^a/a^* cells was determined by flow cytometry.

Due to the short life span of blood cells, their continuous production depends on hematopoietic stem cells, as only they are able to sustain life-long self-renewal and differentiated progeny. Different reconstituting cells can be distinguished from each other based on the durability of engraftment. Whereas short term and intermediate term reconstituting cells are not able to produce differentiated progeny for life, long term reconstituting cells are characterized by their ability to permanently reconstitute the bone marrow [4], [5]. In a lethally irradiated host animal, long term engraftment depends on long term reconstituting cells from the donor, with all blood lineages originating from the transplanted donor stem cell population.

**Figure 1:**
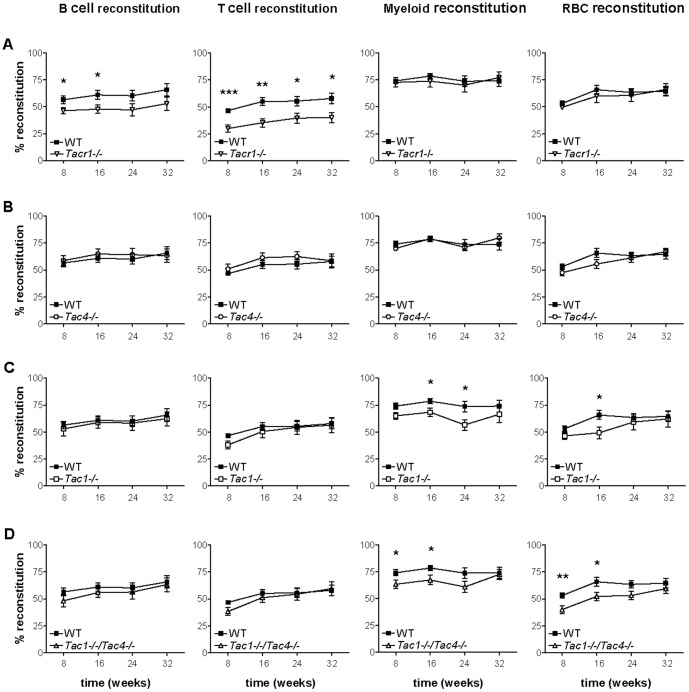
Analysis of the *in vivo* reconstitution efficiency of tachykinin knockout and wild-type mice. *In vivo* reconstitution efficiency for **(A)** *Tacr1* ^−/−^ (NK-1R ko mice, n = 10), **(B)** *Tac4* ^−/−^ (HK-1 ko mice, n = 8), **(C)** *Tac1* ^−/−^ (SP/NKA ko mice, n = 7) and **(D)** *Tac1* ^−/−^/*Tac4* ^−/−^ (SP/NKA/HK-1 double ko mice, n = 8) versus C57BL/6 mice (n = 10). Each graph shows four time points (8, 16, 24 and 32 weeks) when blood was drawn to assess the donor/host reconstitution efficiency by flow cytometry. Four independent *in vivo* reconstitution experiments were performed and three to five individual recipient mice per strain were injected in each experiment. The results of the four experiments were pooled. Knockout mouse strains versus wild-type were analyzed at each time point separately using Student's t-test. Time points where knockouts differ significantly from wild-type are indicated and defined as follows: *p = 0.01–0.05, **p = 0.01–0.001 and ***p<0.001.

Our long term engraftment results show that when bone marrow cells taken from *Tacr1* ^−/−^ mice were tested in a competitive setting, we detected lineage-specific engraftment deficits in NK-1R deficient cells as they reconstituted B and T cell populations to a significantly lower level than the competitor (Fig. 1A). Myeloid and red blood cell repopulation efficiencies were not altered. Since a defective hematopoietic stem cell would affect all lineages equally, this suggests a defect or disadvantage of NK-1R deficient B and T precursor cells. Splenic composition (donor versus competitor) was also examined in mice reconstituted with a mix of NK-1R^−/−^ and wild-type cells, and yielded a similar decrease in NK-1R^−/−^ B and T cells versus wild-type cells (data not shown).

In order to test whether the lack of tachykinin peptides would lead to a similar phenotype, we included mice deficient for SP and/or HK-1 (*Tac4* ^−/−^, *Tac1* ^−/−^ and *Tac1* ^−/−^/*Tac4* ^−/−^ mice) in our long-term bone marrow repopulation experiments. We show that *Tac4* ^−/−^ bone marrow and competing wild-type bone marrow contributed equally to engraftment of mature B, T, myeloid and red blood cells (Fig. 1B). Bone marrow from *Tac1* ^−/−^ and *Tac1* ^−/−^/*Tac4* ^−/−^ mice contributed equally to mature B and T cell populations, but the lack of SP seemed to have a minor and transient effect on the engraftment of the erythromyeloid lineage (Fig. 1C and D). Despite the recently reported bone marrow B cell phenotype of *Tac4* ^−/−^ mice which showed increased pro B cell numbers [15], and the reported effects of SP and NKA on bone marrow hematopoiesis [12], [13], neither *Tac4* ^−/−^, *Tac1* ^−/−^, nor *Tac1* ^−/−^/*Tac4* ^−/−^ mice lacking SP, NKA and HK-1 exhibited B and T cell engraftment deficits similar to *Tacr1* ^−/−^ bone marrow. Since tachykinin peptides are secreted by both surviving host cells and by the competitor bone marrow cells, this result is not surprising as any defect caused by the lack of tachykinin peptides in the donor animal may be compensated by tachykinin secreting cells in the host. Our study therefore suggests a non-autocrine tachykininergic signalling pathway and we conclude that tachykinins released by the host cells or the competitor cells compensate for the inability of the donor cell to produce tachykinin peptide. However, since our results demonstrate B and T cell engraftment deficits in *Tacr1* ^−/−^ mice, this indicates a role for tachykinins in hematopoiesis.

To follow up on the decreased B and T cell reconstitution efficiency of *Tacr1* ^−/−^ derived bone marrow, we performed a thorough analysis of *Tacr1* ^−/−^ primary lymphoid organs using flow cytometry. However, all B, T and myeloid populations were accounted for and no major deficits could be detected, suggesting that B, T and myeloid development is not altered in these mice (Table 1). Furthermore, *in vitro* development of *Tacr1* ^−/−^ bone marrow cells was similar to wild-type controls as their response to IL-7, GM-CSF, IL-4, GM-CSF+IL-4 and M-CSF were comparable (data not shown). In addition, analysis of IL-7 cloning efficiency, which is used to determine the frequency of IL-7 responsive B cell precursors in bone marrow, was similar between *Tacr1* ^−/−^ and wild-type bone marrow (data not shown). No differences in T cell development were observed between *Tacr1* ^−/−^ and wild-type controls when bone marrow progenitors were co-cultured on OP9-DL1 cells at all time points analysed (Table 2).

**Table 1:** Flow cytometry analysis of bone marrow and thymus of C57BL/6 and *Tacr1* ^−/−^ mice.

			C57BL/6	*Tacr1* ^−/−^
**Bone marrow**			%	%
Gated on CD19^+^ cells	CD43^+^B220^+^	pro B	17.1±1.2	19.0±1.0
	CD43^−^B220^low^	pre/immature B	55.1±2.0	49.2±2.0
	CD43^−^B220^high^	mature B	23.6±1.6	26.7±2.2
	IgD^−^IgM^+^	immature B	13.0±0.7	12.5±1.0
	IgD^+^IgM^+^	mature B	22.6±1.8	25.8±2.0
Gated on live cells	CD11b^+^Gr1^+^	myeloid	28.9±1.4	30.6±2.0
Gated on CD3^+^cells	CD4^+^CD8^−^	CD4^+^ T	19.6±1.3	18.7±1.1
	CD4^−^CD8^+^	CD8^+^ T	31.3±1.9	35.2±1.5
**Thymus**				
Gated on lymphocytes	CD8^−^CD4^−^	DN	3.4±0.3	5.0±1.1
	CD8^−^CD4^+^	CD4^+^	10.2±0.6	10.1±0.6
	CD8^+^CD4^−^	CD8^+^	4.5±0.4	5.6±0.5
	CD8^+^CD4^+^	DP	81.9±0.9	79.3±1.7
Gated on CD3^+^ cells	CD8^−^CD4^−^	DN	3.5±0.3	3.3±0.4
	CD8^−^CD4^+^	CD4^+^	59.3±1.7	57.3±1.0
	CD8^+^CD4^−^	CD8^+^	20.4±0.9	22.0±0.6
	CD8^+^CD4^+^	DP	16.8±1.1	16.8±0.6
Gated on CD4^−^CD8^−^	CD25^+^CD44^−^	DN1	29.7±1.1	31.5±1.5
	CD25^+^CD44^+^	DN2	8.4±1.1	7.7±0.6
	CD25^−^CD44^+^	DN3	16.1±1.0	17.8±1.2
	CD25^−^CD44^−^	DN4	45.8±1.4	42.1±0.7

Cells were stained with a combination of antibodies commonly used for the analysis of immune cells. Flow cytometry was performed on FACSCalibur (BD Biosciences, Mississauga, Canada) and data analysis was conducted using Cell Quest Pro. All experiments were performed using female mice, 8–12 weeks old. Each experiment included age-matched sets of knockout and wild-type mice. Statistical analysis was performed using Prism Graph Pad. Student's t-tests revealed no significant differences in total cellularity or in cell populations between C57BL/6 and *Tacr1* ^−/−^ mice. Mean cell number (×10^6^) for bone marrow and thymus respectively were: C57BL/6 (n = 10) 79.2±5.6, 168.1±17.2 and *Tacr1* ^−/−^ (n = 11) 90.4±4.3, 125.7±15.1. Data are presented as mean of % gated±SE.

**Table 2:** T cell development on OP9-DL1 cells.

	day 7	day 10	day 14	day 18
*Tacr1^−/−^* CD44^+^ CD25^+^	48.0±4.3	33.0±15.0	8.0±2.7	1.6±0.4
WT CD44^+^ CD25^+^	51.0±2.7	30.0±14.8	8.3±4.5	2.8±1.2
*Tacr1^−/−^* CD44^−^ CD25^+^	9.7±1.8	63.0±14.6	87.3±3.0	74.0±18.5
WT CD44^−^ CD25^+^	12.3±6.7	61.0±9.2	84.0±3.9	84.0±6.0
*Tacr1^−/−^* DP			0.5±0.4	14.0±8.2
WT DP			2.4±1.4	26.0±3.5

Hematopoietic stem cells (Lin^−^ CD117^+^ Sca1^+^) isolated from bone marrow were co-cultured with OP9-DL1 cells and analyzed by flow cytometry. The proportion of DN2, DN3 and DP cells gated on CD45^+^ are shown on days 7, 10, 14 and 18. Analysis for the expression of CD44 and CD25 were additionally gated on CD4^−^ CD8^−^ CD11b^−^ CD19^−^ and B220^−^ cells. Data are derived from 2–3 independent experiments. All experiments were performed using female mice, 8–12 weeks old. Each experiment included age-matched sets of knockout and wild-type mice. Statistical analysis was performed using Prism Graph Pad. Student's t-tests revealed no significant differences in cell populations between wild-type and *Tacr1* ^−/−^ mice.

While the lack of a phenotype in primary immune organs of *Tacr1* ^−/−^ mice suggests that NK-1R signalling is dispensable for hematopoietic function *in vivo*, the significantly decreased B and T cell engraftment of *Tacr1* ^−/−^ versus wild-type bone marrow is intriguing. One explanation for the lower efficiency of B/T cell engraftment of *Tacr1* ^−/−^ hematopoietic stem cells and/or consequently its predecessors could be a slower cell proliferation rate in the absence of tachykinin signalling. Both wild-type host and competing bone marrow cells express SP and HK-1, which have been shown to positively affect cell proliferation. Therefore, in a competitive setting, wild-type cells receiving signalling through NK-1R may proliferate faster than NK-1R deficient cells. However, the lack of an immunological phenotype in primary lymphoid organs together with the fact that *Tacr1* ^−/−^ mice are capable of repopulating B and T cells *per se*, suggests that NK-1R signalling does not play a key role in hematopoiesis, but rather plays a minor modulating role as one of many signalling pathways supporting the proliferation of B and T cells.

## Supporting Information

Figure S1 **Results of genotyping of litters of mice of various mouse strains.** In order to maintain the integrity of the C57BL/6 background across all of our strains we routinely cross our gene deleted mice to C57BL/6 WT mice and select knockout breeders. Fig. S1 shows such an analysis. This procedure was carried out throughout the course of these experiments, both before and after the reported data. PCR genotyping was carried out using the REDExtract-N-Amp™ Tissue polymerase chain reaction (PCR) kit (Sigma-Aldrich Inc., St. Louis, MO, USA). This figure shows data for three of the mouse strains reported in this manuscript (*Tac1* ^-/-^, *Tac4* ^-/-^, and *Tacr1* ^-/-^). The analysis for the 4^th^ strain, double knock out *Tac1* ^-/-^/*Tac4* ^-/-^, was undertaken at the same time but has already been published [17]. The primers used to genotype the four knockout strains have been previously reported as well [17].
(TIF)
