# Supplementary figures and images for: Neurokinin-1 Receptor Signalling Impacts Bone Marrow Repopulation Efficiency
Source: PLoS One. 2013 Mar 14;8(3):e58787. doi: 10.1371/journal.pone.0058787 (PMC3597582; doi:10.1371/journal.pone.0058787)

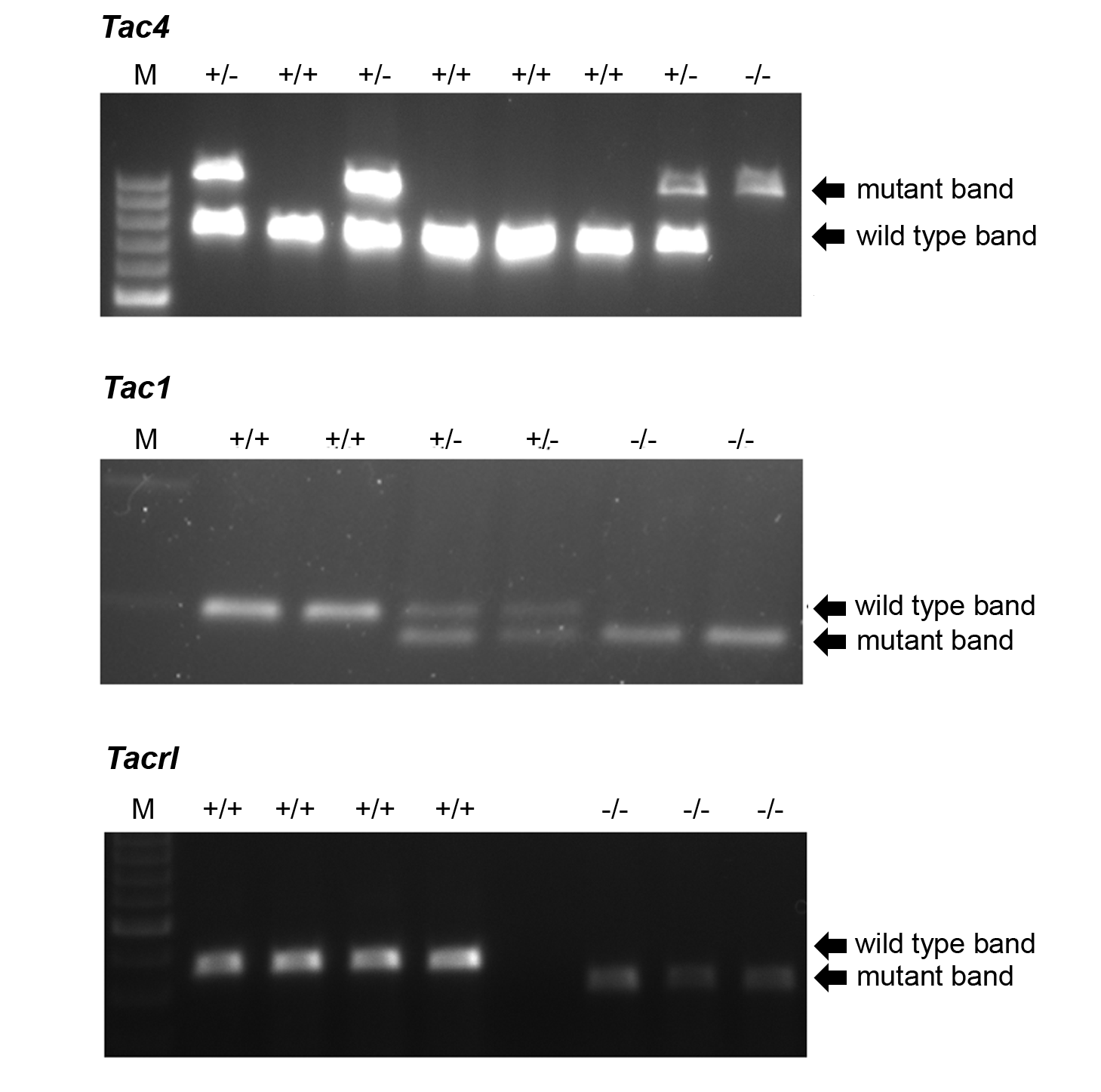

Supplement: Figure S1 — Results of genotyping of litters of mice of various mouse strains. In order to maintain the integrity of the C57BL/6 background across all of our strains we routinely cross our gene deleted mice to C57BL/6 WT mice and select knockout breeders. Fig. S1 shows such an analysis. This procedure was carried out throughout the course of these experiments, both before and after the reported data. PCR genotyping was carried out using the REDExtract-N-Amp™ Tissue polymerase chain reaction (PCR) kit (Sigma-Aldrich Inc., St. Louis, MO, USA). This figure shows data for three of the mouse strains reported in this manuscript (Tac1 -/-, Tac4 -/-, and Tacr1 -/-). The analysis for the 4th strain, double knock out Tac1 -/-/Tac4 -/-, was undertaken at the same time but has already been published [17]. The primers used to genotype the four knockout strains have been previously reported as well [17]. (TIF) [file pone.0058787.s001.tif]
